# System for continuous metabolic monitoring of mechanically ventilated patients

**DOI:** 10.3389/fmed.2024.1356087

**Published:** 2024-07-02

**Authors:** Gary Shaw, Francesco Vicario, Roberto Buizza

**Affiliations:** ^1^Lincoln Laboratory, Massachusetts Institute of Technology, Advanced EO Systems, Lexington, MA, United States; ^2^Philips, Eindhoven, North Brabant, Netherlands

**Keywords:** mechanical ventilation, indirect calorimetry, continuous metabolic tracking, enteral nutrition, prototype

## Abstract

In clinical settings, due largely to the cost, size and calibration complexity of existing indirect calorimetry systems, there is seldom instrumentation available to provide reliable, continuous tracking of a mechanically ventilated patient’s metabolic output in support of proper nutrition. The atypical metabolisms associated with critically ill patients are difficult to predict and both underfeeding and overfeeding lead to negative impacts on both mortality and the recovery and healing processes. With these issues in mind, a novel ventilator-agnostic indirect calorimetry sensor design, prototype development, and validation were undertaken with the goal of enabling 24/7 metabolic monitoring of mechanically ventilated patients by means of a passive, rate-proportional side-stream sampling scheme and miniature mixing chamber. The miniature mixing chamber enables the use of small, low-cost gas concentration and flow sensing components to ensure the affordability of commercial design-for-manufacture implementations of the prototype sensor. In addition to continuous measurement of patient metabolism, the prototype sensor also enables autonomous monitoring and detection of calibration drift in the gas measurement sensors without disrupting the patient ventilation.

## 1 Introduction

This paper presents the architecture and prototype implementation of a ventilator-agnostic indirect calorimetry (IC) system to track the energy expenditure and fuel substrate utilization of mechanically ventilated patients. The prototype architecture leverages elements of a patented personal metabolic sensor, the Carbon-dioxide and Oxygen Breath and Respiration Analyzer (COBRA) (1–4), modified to accommodate the higher levels of O_2_ encountered in a mechanically ventilated patient. Accuracy of the prototype system over a range of ventilator settings is quantified by means of a simulation test bench.

### 1.1 Motivation for continuous metabolic monitoring of ventilated patients

For an individual in the intensive care unit (ICU) on a mechanical ventilator, the question “What should we feed this patient and how much?” is extremely important for optimal recovery, and is a challenge to answer accurately in a clinical setting. A common approach employed to determine a ventilated patient’s energy needs is to have a dietitian employ predictive equations derived from data for a general population. In general the various estimators rely on parameters such as height, weight, body mass index, age, and gender. This approach falls short with patients not represented by the population norms used to derive the predictive equations, which is often the case, especially for the acutely ill (5–7). The presence of injury or acute illnesses in hospitalized patients alters the metabolic response and makes predictive equations inadequate, as first shown in a seminal study by Long et al. (8) where, for example, it was found that fever and infection increase energy expenditure by up to 80%. Mechanically ventilated patients have better medical outcomes when their diet is tailored to properly meet both caloric and macronutrient needs. The atypical metabolisms associated with critically ill patients are difficult to predict and both underfeeding and overfeeding lead to negative impacts on both mortality and the recovery and healing processes. Mault (9) showed that a cumulative energy deficit, implying that the calories delivered may not be equal to the calories metabolized by the patients, is associated with increased days on mechanical ventilation and a longer overall ICU stay. Silva et al. (10) showed a correlation between enteral nutrition discontinuation and mortality for hospital patients. Similarly, deleterious effects are associated with significant overfeeding including poor glycemic control, altered neuroendocrine responses, increased risk of infectious complications, delayed liberation from mechanical ventilation, and even increased mortality (11, 12).

Indirect calorimetry, based on quantitative analysis of respired breath, is a proven means of determining metabolic energy and fuel substrate mix for a subject. The volume rates of CO_2_ produced (VCO_2_), and O_2_ consumed (VO_2_), provide an indication of what mix of chemical reactions are occurring on a cellular level and, in the case of a resting measurement, what macronutrients are being metabolized. Further, by comparing the measured energy expenditure and macronutrient utilization of the patient to the dietary intake, a determination can be made of whether the macronutrients being metabolized are being supplied by the nutrient feeding regimen or by the patient’s own fat/glycogen stores, or skeletal muscle.

A 2016 study by Rousing et al., of seven different methods employed for parametric estimation of energy expenditure in the absence of indirect calorimetry (IC) found that the various estimation methods achieve an error of 10% or better less than half the time, typically only about 30% of the time (13). The 2016 study suggested VCO_2_, with an assumed respiratory quotient (RQ), as a cost-effective means of approximating true IC. However, the study also showed VCO_2_-only IC results in higher sensitivity to variations in minute volume (13). In addition, VCO_2_-only IC provides no insight into fuel substrate utilization. A 2023 study found the rate of malnutrition in mechanically ventilated COVID patients was remarkably high and largely undocumented. Most patients did not receive the minimum estimated protein needs (14).

In addition to providing the metrics necessary to tailor the patient’s nutrition regimen, tracking changes in energy expenditure, VO_2_, and substrate mix over time may also provide an early indication of changes in the medical condition of the patient and thus aid in refining and confirming a proper diagnosis and treatment (15–17).

### 1.2 Historical barriers to continuous metabolic monitoring

At the time of this writing, 8 years since the publication of the Rousing study, it is still the case that in clinical settings there is seldom instrumentation available to the attending physicians to enable reliable determination of a mechanically ventilated patient’s metabolic output. Further, the patient is typically non-communicative and unable to express feelings of either hunger or satiety. While commercial systems are available for making the necessary measurements to characterize the patient’s metabolic state, the lack of use in the ICU seems to be a consequence of several factors including:

- System size and procurement costs of $20 K or more
- Complexity and time required for setup, calibration, and operation
- Inability of existing systems to function for days or weeks while providing continuous measurement and automated analysis of patient energy expenditure, substrate oxidation mix, and metabolic efficiency

While spot measurements of energy expenditure and fuel substrate may be made using a system such as the Cosmed QNRG+, a filter and spirometer must be inserted in the patient Y, so named because it joins the inhale and exhale limbs of the ventilator at the mouth of the patient. Furthermore, the duration of the data collect is limited to 30 min or less before clogging of the heat moisture exchange (HME) filter becomes an issue (18). More importantly, nurses and physicians have neither the time nor software tools to interpret hours or days of metabolic data in order to tailor a patient’s dietary regimen. Therefore, to realize the benefits continuous 24/7 metabolic monitoring, machine processing of the data to produce and track key metabolic parameters such as energy expenditure, fuel substrate, VO_2_ and VCO_2_, is required in order to accurately tailor feeding regimens to the patient’s unique evolving metabolic needs. Furthermore, automatic checking of gas sensor calibration without disruption of the mechanical ventilation is desirable to ensure accuracy is maintained over the days or weeks of continuous measurement.

## 2 Ventilator agnostic indirect calorimeter design philosophy

This section explores architectural design trades aimed at addressing the issues of cost, continuous monitoring, ease of use, and assurance of gas sensor calibration accuracy. The design trades include one vs. two mixing chambers, the ability to swap mixing chambers between ventilator inhale and exhale limbs, and mechanisms for confirming gas concentration and flow sensor accuracy.

### 2.1 Overarching objectives

Given the historical barriers to widespread use of metabolic sensing for mechanically ventilated patients, the primary objectives driving the prototype sensor development include

1. Capability to provide continuous 24/7 metabolic monitoring of mechanically ventilated patients over the range of ventilator oxygen and respiration-mode settings encountered in a clinical setting
2. An architecture enabling the use of slow, hence low-cost, gas concentration and flow sensing components to ensure affordability of a design-for-manufacture (DFM) implementation of the IC sensor
3. A ventilator-agnostic architecture to ensure widespread compatibility
4. Capability to monitor and detect calibration drift in the gas sensors without disrupting the patient ventilation
5. Capability to quickly replace an out of calibration or faulty sensor without disrupting the patient ventilation
6. Data processing options to enable cancellation of bias in gas sensor measurements
7. Data processing algorithms to provide VO_2_, VCO_2_, energy expenditure, respiratory exchange ratio (RER) and, from these metrics, determine optimal caloric and macronutrient feeding regimens

### 2.2 Leveraging existing personal metabolic sensor technology

**Figure 1:**
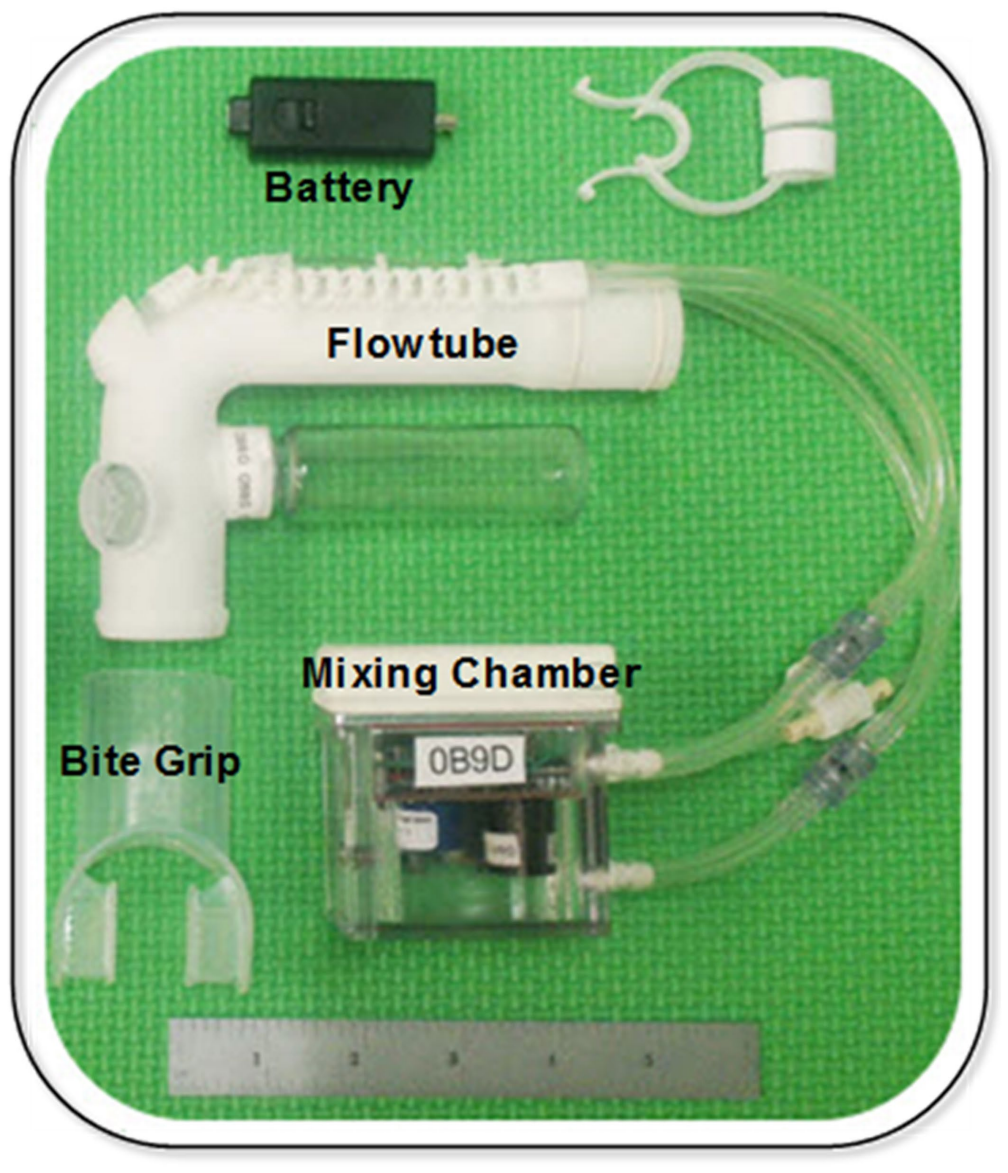
COBRA prototype sensor (18).

As noted in the introduction, the starting point to develop a small low-cost, ventilator-agnostic indirect calorimeter capable of continuous metabolic monitoring of ventilated subjects was a previously designed, prototyped and validated metabolic sensor for personal on-demand indirect calorimetry. The Carbon-dioxide and Oxygen Breath and Respiration Analyzer (COBRA) sensor needs no special training to use, is wireless and hand-held, making it extremely portable and suitable for activity levels from resting to intense exercise (19–21). A central theme in the design of the COBRA sensor was to implement a miniature mixing chamber for gas measurement in order to achieve small size and weight and employ slow, and hence inexpensive, gas sensors. Commercially available indirect calorimetry sensors designed to miniaturize or eliminate the mixing chamber altogether employ one of two strategies. By employing high-speed (25–50 Hz) sensors to perform sequential measurement of flow and gas concentrations, followed by time alignment and integration of the differential measurements the need for a mixing chamber can be eliminated (22, 23). However, this breath-by-breath technique requires high-speed, and therefore costly, flow and gas sensors as well as rigorous calibration to ensure time alignment of the sequential measurements. An alternative strategy is to employ high-speed breath sensing with active switching of valves to divert a flow- rate proportional fraction of exhaled gas to a miniature mixing chamber (24). In comparison, the hands-free COBRA prototype sensor (19) achieves the required flow-rate-proportional side-stream sampling by means of the patented passive flowtube design and miniature mixing chamber pictured in Figure 1.

The challenges faced in adapting the COBRA personal metabolic sensor for use in a clinical setting with mechanically ventilated patients include:

- Accommodating intubated, non-responsive patients
- Accommodating supplied oxygen levels that are variable up to 100% O_2_
- Accommodating ventilator gas flow profiles that may differ substantially from those of unventilated, healthy subjects including positive end expiratory pressure (PEEP)

### 2.3 Rate-proportional, passive side-stream sample port design and location

From a signal-to-noise perspective, the patient Y, so named because it joins the inhale and exhale limbs of the ventilator at the mouth of the patient, is the best location for a metabolic sensor to sample since it provides direct access to the patient inhale and exhale flow rates and gas concentration, while excluding the dilution effects of ventilator blow-by flow. In fact, this is the location that existing metabolic sensors designed to make measurements of ventilated patients, employ.

However, in terms of other performance metrics, there are significant disadvantages of sampling in the patient Y, as compared to the ventilator inhale and exhale limbs. One disadvantage of sampling at the patient Y is that the flow is bidirectional and therefore requires suppression of sampling during inhale to avoid dilution of the exhaled mixing chamber gases. This can be accomplished by employing a high temporal resolution flow sensor to control a pulse-width modulated mechanical valve in conjunction with a constant rate pump to achieve rate-proportional sampling on exhale and to suppress sampling on inhale. The duty factor is driven by the instantaneous flow profile and requires a high temporal resolution flow sensor and a fast valve. Details of the technique are described in Brugnoli and Laziale (24).

The COBRA architecture avoids the need for fast gas sensors, valves and active pumps by means of a uniquely designed flowtube that supports passive, rate-proportion, valveless sampling and also behaves as a flow diode, inhibiting flow into the mixing chamber during inhale. As described in Shaw et al. (20), during exhale, a venturi in the flowtube creates sufficient flow resistance to divert ~1% of the mainstream exhale flow into the mixing chamber in proportion to the flow rate. The sample displaces an equal amount of well mixed gas in the chamber and returns it to the mainstream exhale path. During inhale, the location and orientation of the sample and return ports in the flowtube inhibits the flow of inhale gas into the mixing chamber. This flow-rate proportional sampling during exhale, and diode-like behavior of the flowtube during inhale, eliminates the need for a pump or active valve. The COBRA venturi design also enables measurement of flow by means of a commercial off-the-shelf (COTS) differential pressure sensor than monitors the pressure drop across the venturi.

**Figure 2:**
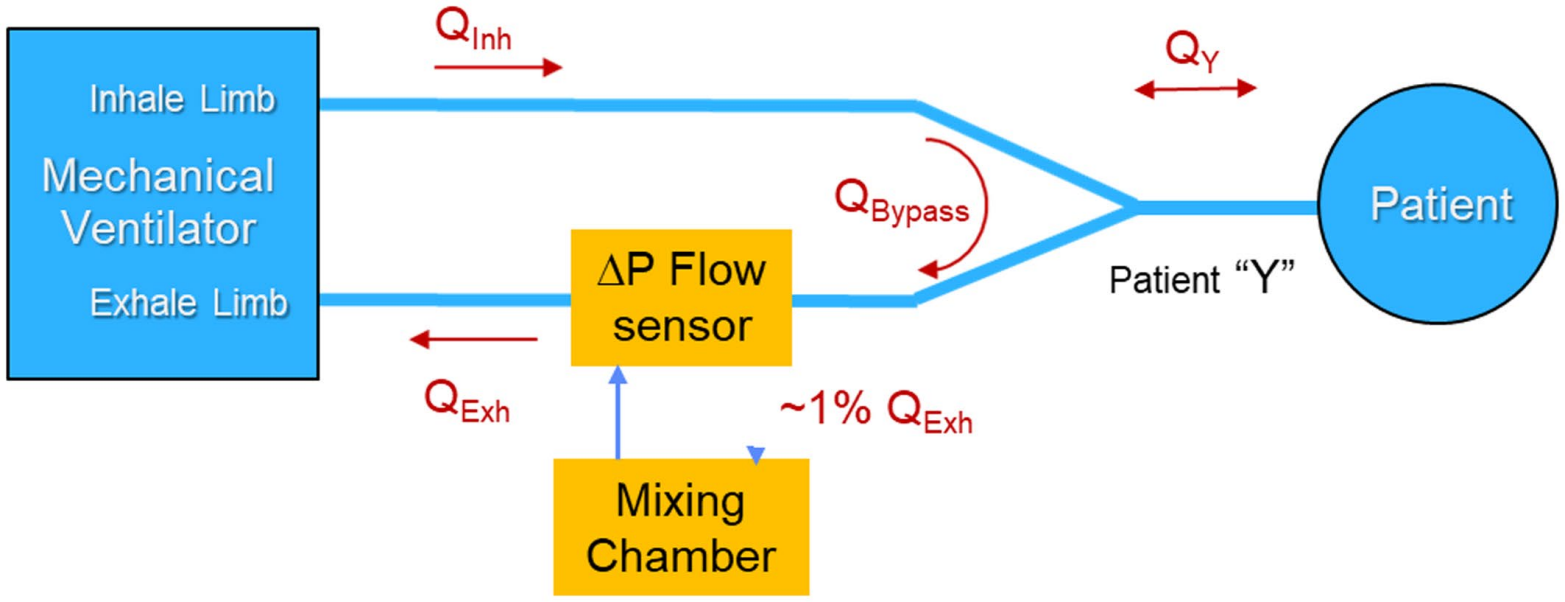
Nominal location of the COBRA metabolic sensor in the mechanical ventilator circuit.

However, placing the COBRA flowtube and associated sample ports in the patient Y increases dead space, which reduces the efficacy of ventilatory support, and adds respiratory burden, sample tubes, and additional weight to the patient Y. These negative attributes of sampling at the patient Y are tolerable, but not desirable, and even less so when aiming to monitor metabolism continuously, rather than via infrequent, spot-check measurements. Consequently, it was decided to locate the sample port that feeds the mixing chamber in the exhale limb of the ventilator as illustrated in Figure 2 rather than in the patient Y. Table 1 summarizes the performance trades associated with the location of the the sample port.

**Table 1:** Summary of the performance trades associated with location of the sample ports with blue font indicating the advantage.

Parameter	Patient Y	Exhale limb	Explanation
Flow direction	Bidirectional	Unidirectional	Unidirectional flow simplifies gas sampling proportionality
Volume flow rate	Tidal volume only	Tidal volume + blow-by	By sampling in the exhale limb, the mixture of patient exhale plus blow-by gas dilutes the exhale concentration thereby reducing the signal-to-noise ratio presented to the volumetric gas sensors.
Mixing of exhaled tidal gas volume	Lowest	Highest	By sampling further away from the Y and closer to the ventilator exhalation port, gas is pre-mixed along the exhalation tube. This reduces the negative impact that deviations from sampling proportionality have on the final VO_2_ and VCO_2_ measurements.
Added dead space	~12 cc	None	Dead space reduces the effectiveness of ventilation therapy
Respiratory burden	Inhale and exhale	Exhale only	Added respiratory resistance at the patient Y reduces the effectiveness of ventilation therapy
Weight burden	~20–30 g	None	
Obstructed vision	Sample tubes	None	
Proximity to electronics	Distant	Close	

A major advantage of placing the sample port in the exhale limb is that the flow is always uni-directional, and therefore there is no need for the sample port to behave as a flow diode. By placing the sample port near the ventilator, far from the patient Y, a further benefit can be realized by taking advantage of the mixing that occurs in the corrugated patient ventilator exhale line between the exhale port of the patient Y and the sample port.

The COBRA flowtube was therefore replaced by a commercial flow sensor, designed for specifically for use in the limb of a ventilator, in combination with a custom flow divider designed to adhere to the respiratory burden limit and achieve acceptable flow-rate-proportional, side-stream sampling.

### 2.4 Dual mixing-chamber port-swapping architecture

Provided a measurement of the FiO_2_ on the ventilator inhale limb is available, incorporating a single metabolic mixing chamber on the exhale limb is sufficient to provide the desired VO_2_ and VCO_2_ measurements necessary for determining the respiratory exchange ratio (RER), VCO_2_/VO_2_, and energy expenditure. However, in the prototype it was decided to incorporate identical mixing chambers on both the inhale and exhale limbs of the ventilator and to provide a mechanism for switching the connections of the mixing chambers between the inhale and exhale limbs through a process termed “port swapping.” There are several reasons to incorporate two mixing chambers and implement port swapping, especially during the research stage of the prototyping including:

1. Calibration checks—by swapping mixing chambers without changing ventilator settings or disrupting patient ventilation, the gas measurements from the two mixing chambers on each of the two limbs can be compared with any differences indicating a possible error in calibration. This calibration check can be accomplished without disruption of the patient ventilation.
2. Elimination of bias in the gas sensors—a frequent form of paramagnetic oxygen and CO_2_ sensor error is a zero offset or bias. During periods in which the ventilator settings are unchanged, a potential method of canceling the bias term is to use the gas concentrations from the same mixing chamber measured on the inhale and exhale limbs at two different times, to compute the differential volume fractions of gas. This measurement approach is termed common-mode rejection.
3. Reduction of bias in the flow sensors—Using the Haldane transform, the volume changes in O_2_ and CO_2_ can be referenced to either the inhale flow sensor or the exhale flow sensor and whichever sensor is more accurate will provide a more accurate measure of VO_2_ and VCO_2_. As with the gas calibration check, differences in the VO_2_ and VCO_2_ depending upon which flow sensor is referenced can also provide an indication of bias between the two flow sensors.
4. Tracking gas calibration drift—By employing two mixing chambers and subsequently checking the calibration over time with certified gases, insight can be gained into the calibration stability of the gas sensors and also whether calibration errors are due to individual sensor drift or, if correlated across the sensors, are more likely indicative of an error in the calibration procedure. This information is useful in the prototyping phase to validate the performance of the selected gas sensors and identify alternative sensors if needed.

**Figure 3:**
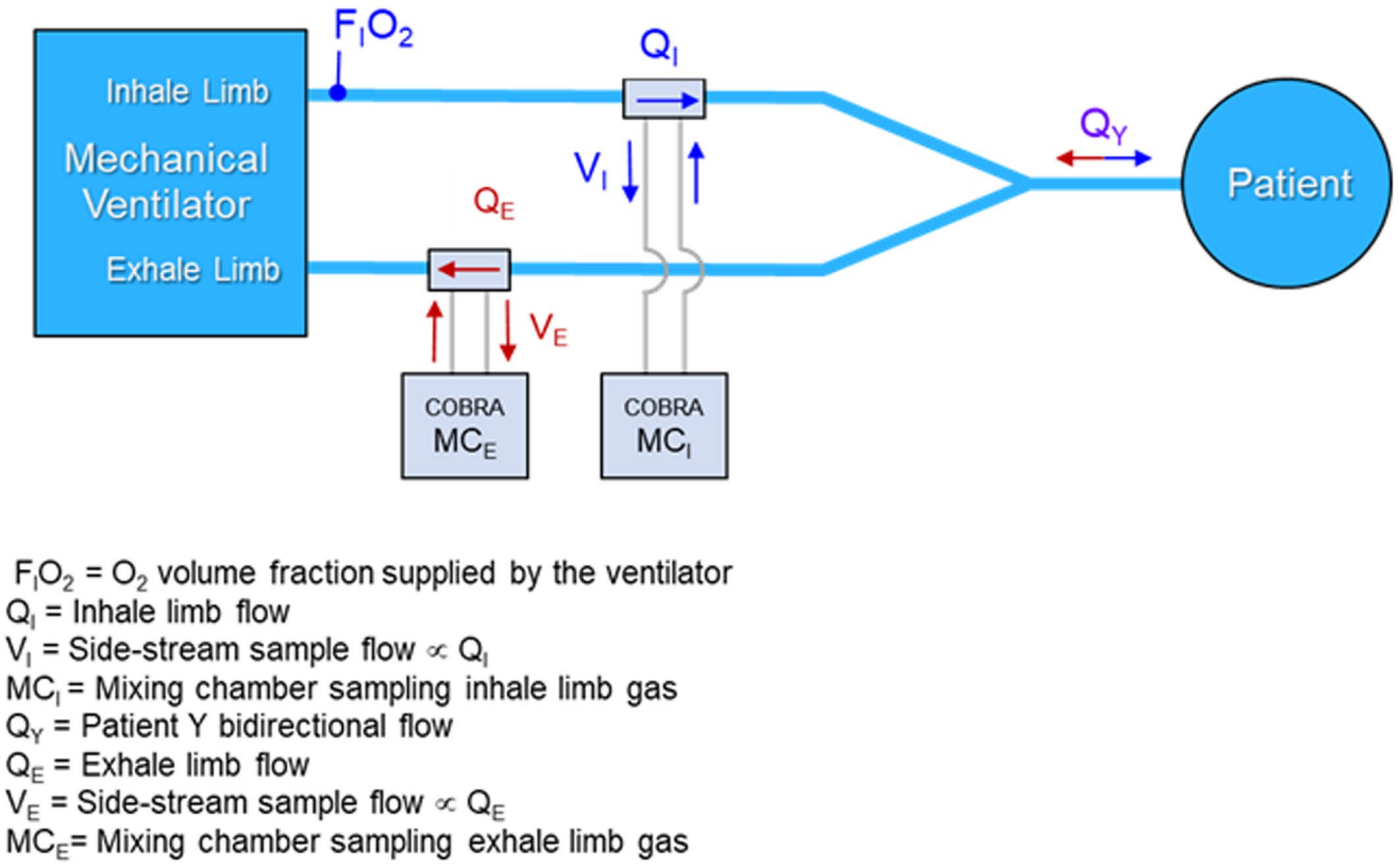
Conceptual diagram of a dual mixing chamber architecture with flow-rate proportional side-stream samples from the inhale and exhale limbs of the ventilator feeding miniature mixing chambers.

While the final architecture of a design for manufacture (DFM) metabolic sensor could conceivably involve only one mixing chamber on the exhale limb plus an O_2_ gas concentration sensor on the inhale limb, the prototype was designed to be more complex, both in terms of the collection and interconnection of hardware, as well as the data collection protocol. Figure 3 is a conceptual block diagram of the dual mixing chamber concept showing connections and flows.

A primary argument for preserving the dual mixing chamber architecture in a DFM implementation of the sensor is the ability to employ port swapping to detect any underlying calibration drift in the gas sensors without disrupting the mechanical ventilation of the patient. This feature could be vital in situations where a patient is on a ventilator for days or weeks, and the importance of metabolic monitoring increases, since calibration might drift but would not otherwise be detectable without disrupting the patient ventilation.

## 3 Prototype system form and function

Given the desired architecture of Figure 3, development of a prototype system suitable for bench testing and validation was initiated with the intent of incorporating as much of the COBRA flow-tube, electronics and mixing chamber design and hardware as possible. Following the naming convention devised for the COBRA sensor, the prototype is dubbed the Carbon-dioxide and Oxygen Respiratory Ventilator Energy Tracker (CORVET). The following subsections describe salient details of the CORVET subsystems and function.

### 3.1 Flow-rate proportional sampling

**Figure 4:**
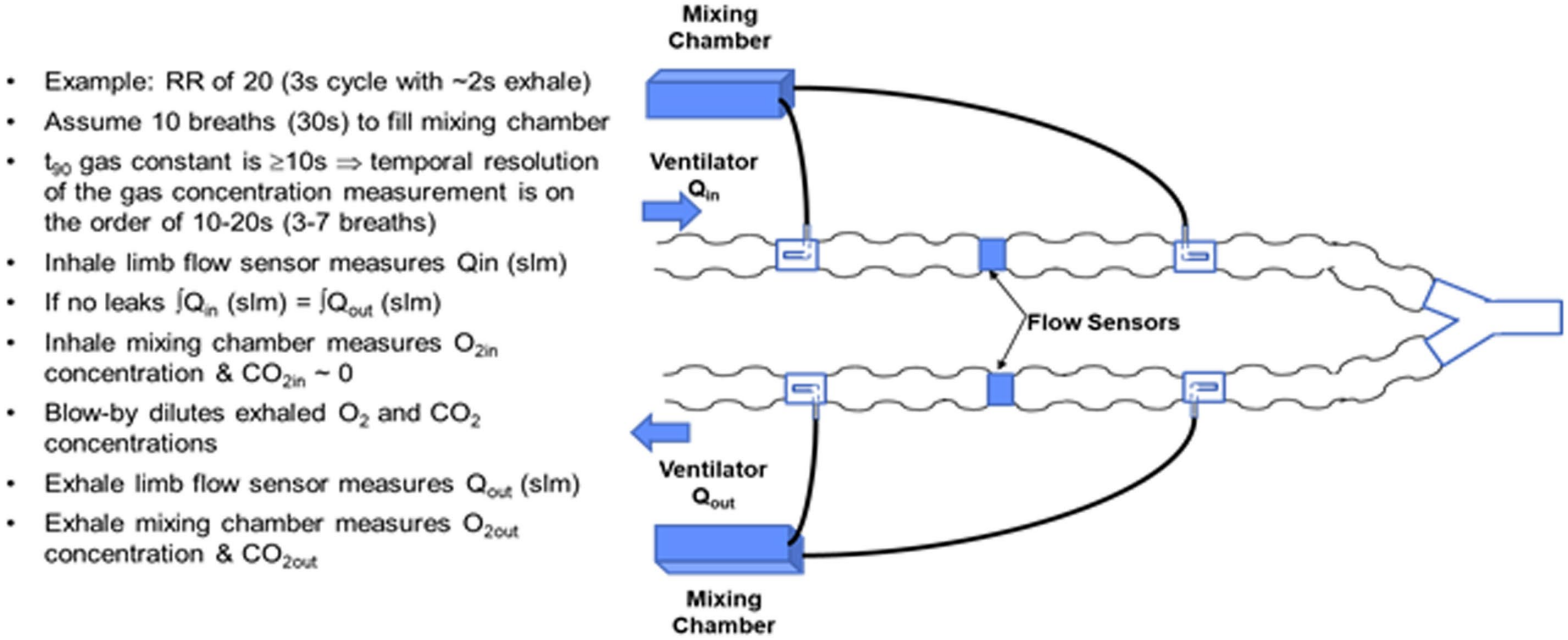
Side-stream rate-proportional sampling architecture employing a commercial flow sensor and custom flow divider design inserted in the input and output limbs of the ventilator circuit.

Abandoning the venturi flowtube design of the COBRA in order to meet respiratory burden limits required conceiving and testing alternative sample port configurations to preserve the flow-rate proportional side-stream sampling of flows in either the inhale or exhale limb of the ventilator, while keeping the respiration burden to less than 0.5 cm H_2_O at a flow rate of 30 LPM. The alternative flow divider concept is based on a pitot tube like configuration in which the proportion of mainstream flow diverted to the mixing chamber is set in part by the cross-sectional area of a pick-off tube facing into the flow relative to the cross-sectional area of the ventilator tube. The flow divider also takes advantage of the differential pressure drop across the COTS flow sensor by sampling ahead of the flow sensor and returning an equivalent volume rate of mixed gas exiting the mixing chamber after the flow sensor as illustrated in Figure 4.

The basic design trades for the flow divider involve the cross-sectional area of the pickoff tube relative to the cross-sectional area of the ventilator tubing, and the flow impedance in the side-stream path. A key to achieving the desired passive, flow-rate proportional, side-stream sampling is to ensure that the flow in the side-stream circuit, specifically laminar versus turbulent, matches the flow in the ventilator limb. During laminar flow, the pressure drop is proportional to the flow rate, whereas during turbulent flow, the pressure drop increases as the square of the flow. The corrugations in the ventilator tube and the tight radius of curvature tend to create turbulent flow, resulting in a high Reynolds number. The side-stream flow divider path must therefore be large enough in diameter and low enough in impedance to also present a high Reynolds number.

**Figure 5:**
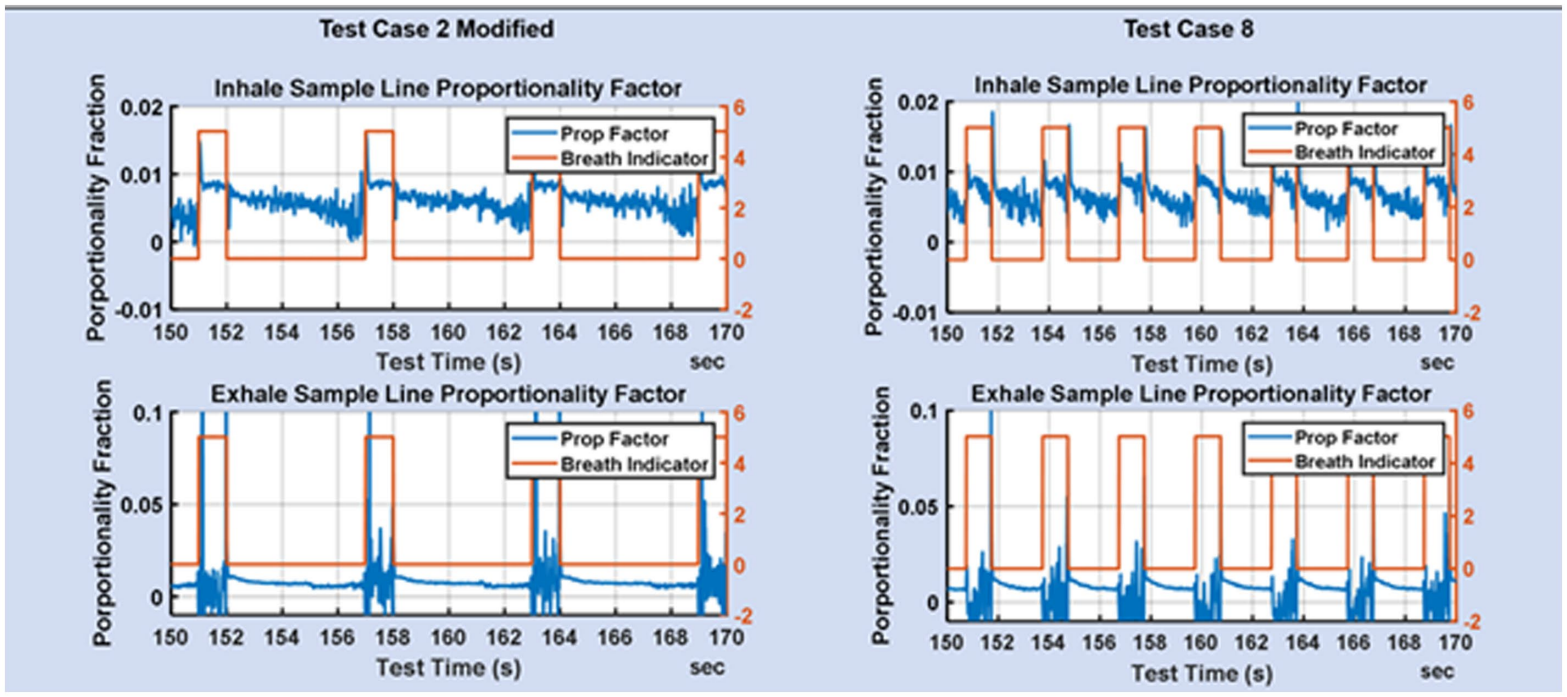
Zoom of the fraction of mainstream flow diverted to the mixing chamber on the inhale limb and exhale limb for TC2 and TC8.

Two blower-only test cases with tidal volume of 0.5 L and respiration rate of 20, were the focus of much of the early flow divider testing. The testing was aimed at quantifying the degree to which rate proportional sampling of the main stream flow was achieved. Representative plots of flow for these two test cases are shown in Figure 5.

Figure 5 shows the proportionality achieved for test cases 2 and 8. The breath indicator goes high during the inhale phase and low during the exhale phase. During inhale, the flow in the exhale limb of the ventilator is low and consequently the flow measurements are noisier, but also of less consequence. During exhale, the flow on the inhale limb is low resulting in noisier flow measurements. The desire is for flow-rate proportionality on the inhale line during inhale, and flow-rate proportionality on the exhale line during exhale. It is not necessary that the proportionality constants be the same on the inhale and exhale lines, since that does not impact the gas fractions measured in the mixing chamber.

### 3.2 Port swapping

**Figure 6:**
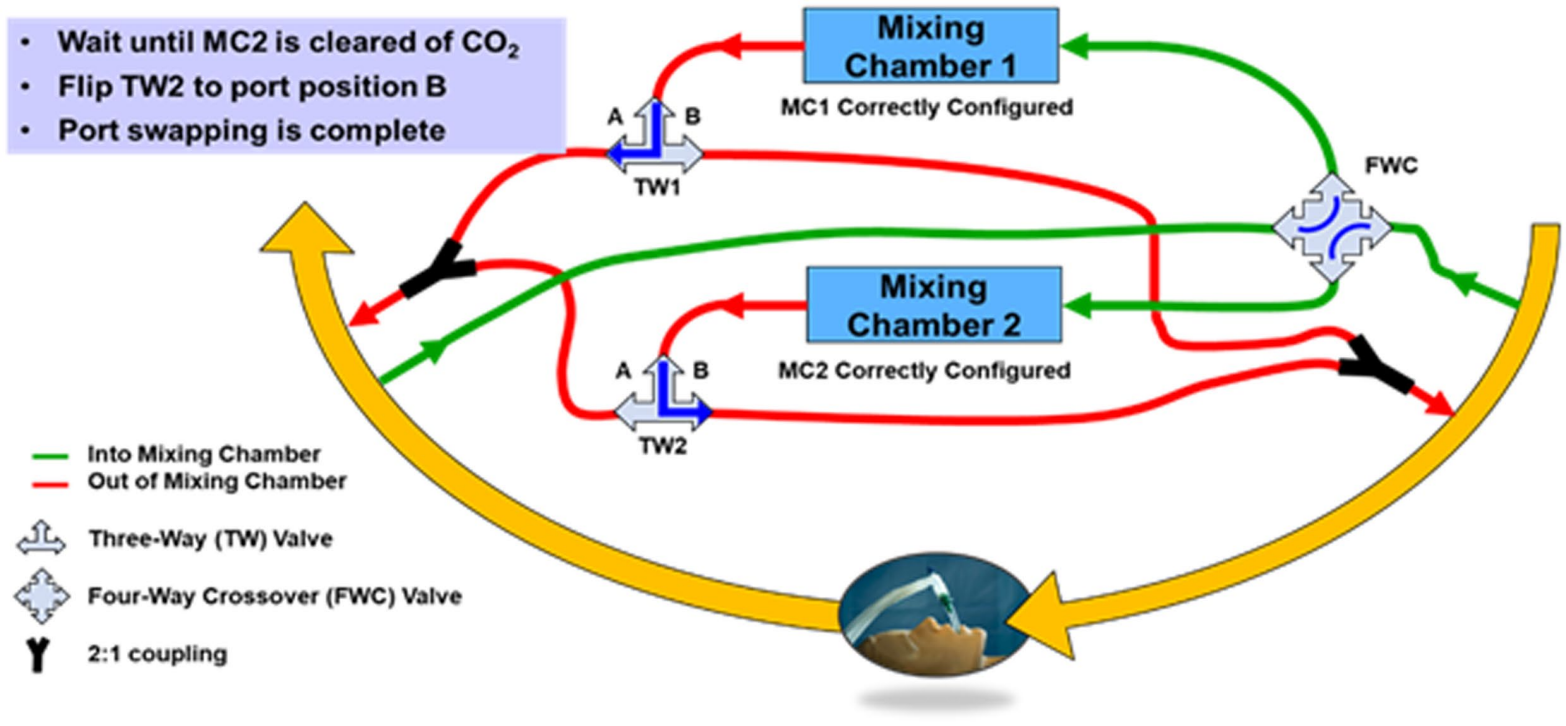
Block diagram of the CORVET dual mixing chamber port-swapping architecture showing mixing chamber 1 sampling inhale limb of the ventilator and mixing chamber 2 sampling the exhale limb of the ventilator.

A conceptual diagram of the side-stream sampling, dual mixing chamber architecture with port-swapping is shown in Figure 6. With reference to Figure 6, the four-way crossover valve enables simultaneous swapping of the mixing chamber input ports between the inhale limb of the ventilator and the exhale limb. However, immediately after a port swap, the mixing chamber that was previously connected to the exhale limb contains exhaled CO_2_ and therefore must remain connected to the exhale limb until a sufficient volume of O_2_ from the inhale limb displaces the CO_2_, at which time the output of the mixing chamber can be switched from the exhale limb to the input limb via the three-way valve.

The conceptual diagram of Figure 6 is clearly more complicated than a single mixing chamber, in part because of the valves and plumbing necessary to allow the mixing chamber connections to be swapped from either inhale or exhale ports. If adopted as a DFM system, the valves for port swapping would be computer controlled and the plumbing designed to minimize required volume. However, for the CORVET prototype, since the benefits and desirability of port swapping were unproven, manually-operated valves were used to avoid investing time and effort into an optimized computer-controlled implementation of port swapping until such time as the value of port swapping was demonstrated.

Key features of the CORVET prototype architecture include

1. Two identical mixing chambers containing CO_2_, O_2_, relative humidity and temperature sensors
2. Two identical passive flow-dividers providing flow-rate proportional side-stream sampling
3. A commercial flow sensor located between the input and output ports of each flow divider
4. Inclusion of a manually-operated four-port crossover valve to enable swapping the input sample port of each mixing chamber to opposite limbs (input vs. output) of the ventilator circuit
5. Two three-way valves to enable swapping the output port of the mixing chamber to opposite limbs (input vs. output) of the ventilator circuit

**Figure 7:**
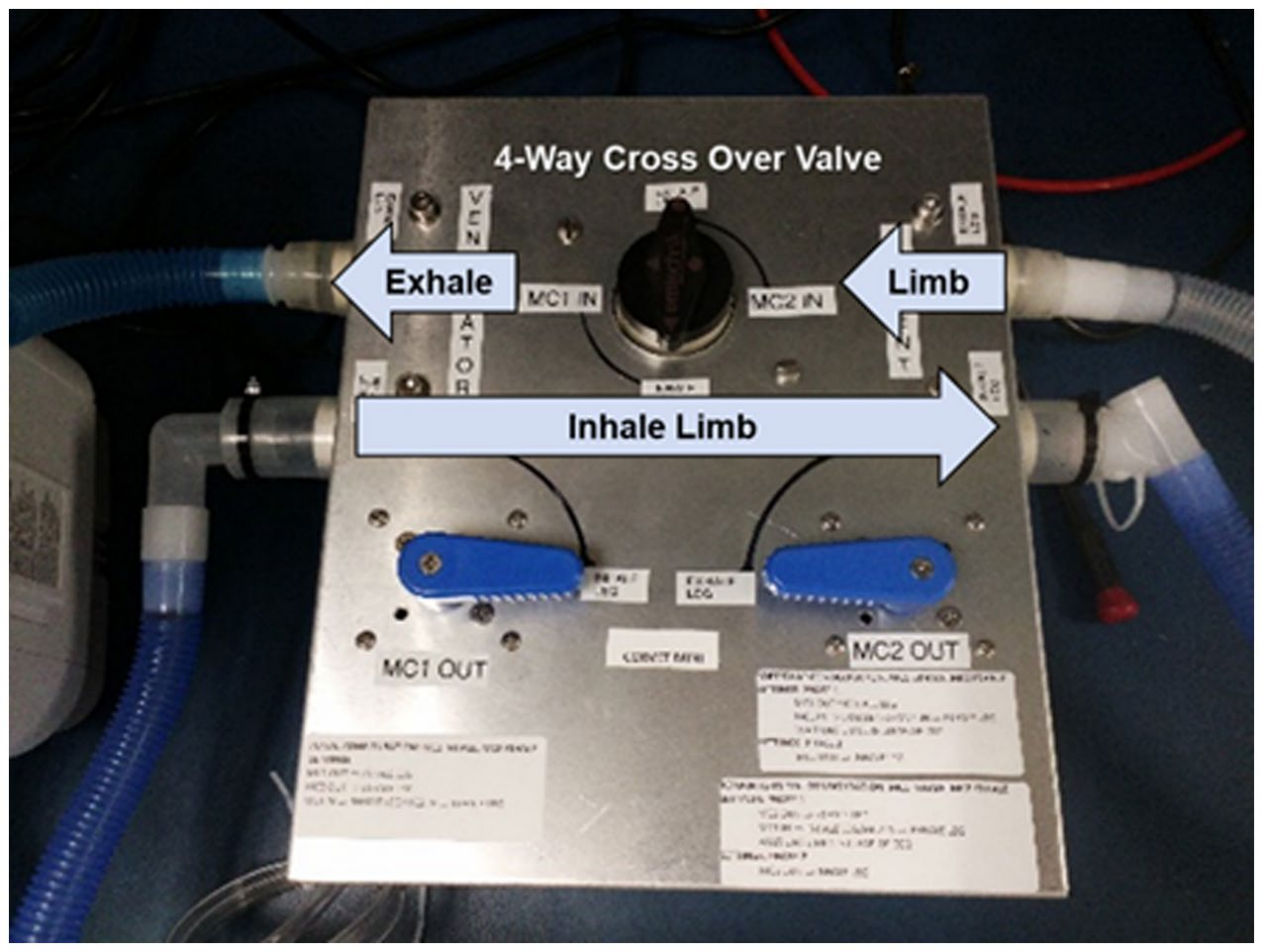
CORVET prototype sensor employing manual valves for swapping mixing chambers between inhale and exhale limbs of the ventilator.

A prototype CORVET sensor was assembled and fitted with a Nafion^®^ drier to enable operation with humidified gases. A photo of the prototype is shown in Figure 7.

### 3.3 Data processing algorithms

The incorporation of two architecturally identical sample ports and mixing chambers on the inhale and exhale limbs of the ventilator supports the formulation of at least five different algorithms for computing VO_2_ and VCO_2_ from the measured data. If the flow and gas sensors were all precise, and there are no leaks in the patient circuit, the simplest method for calculating VO_2_ and VCO_2_ would be to subtract the volumetric flows measured on the inhale limb from those measured on the exhale limb as indicated in Equations 1 and 2.


(1) $$VO_{2}\mathrm{consumed}=Fi{O_{2}}^{*}\mathrm{Qi}–Fe{O_{2}}^{*}Qe$$

(2) $$VCO_{2}\mathrm{produced}=FeC{O_{2}}^{*}Qe–FiC{O_{2}}^{*}\mathrm{Qi}$$

We term this the *direct method* and the errors produced by this algorithm reflect the combined impact of errors in the gas concentration measurements as well as the flow measurements. As expected, the direct method has proven to be the least accurate method for computing VO_2_ and VCO_2_ from the CORVET measurements.

The Haldane transformation is a common method employed in indirect calorimetry to account for the inability to measure the volumetric flow of O_2_ and CO_2_ inhaled. The transformation is based on the assumption that the volume of N_2_ inhaled is equal to the volume of N_2_ exhaled in each breath, as indicated in Equation 3.


(3) $$Fi{N_{2}}^{*}\mathrm{Qi}=Fe{N_{2}}^{*}Qe$$

Consequently, if the volume fraction of N_2_ can be determined for both the inhaled and exhaled air, and the volumetric flow of exhaled air can be measured, the volumetric flow of the inhale is given by


(4) $$\begin{array}{l} \mathrm{Qi}=Qe^{*}\;\left(FeN_{2}/FiN_{2}\right)\\ \;=Qe^{*}\;\left(1-FeO_{2}-FeCO_{2}\right)/\left(1-FiO_{2}-FiCO_{2}\right) \end{array}$$

The O_2_ and CO_2_ inhale concentrations can be measured prior to humidification and used to calculate FiN_2_. In similar fashion, FeN_2_ can similarly be calculated from measured volume fractions of O_2_, CO_2_ and the volume fraction of H_2_O determined from temperature and relative humidity.

Substituting the expression in Equation 4 for Qi, the direct form Equations 1 and 2 yield a set of equations for computing VO_2_ and VCO_2_ that are referenced to the exhale flow only and therefore are not affected by errors in the inhale flow


(5) $$\begin{array}{l} VO_{2}\mathrm{consumed}=Qe^{*}\;\left(Fi{O_{2}}^{*}\left(FeN_{2}/FiN_{2}\right)-FeO_{2}\right)\\ =Qe^{*}\;\left(Fi{O_{2}}^{*}\left(1-FeO_{2}-FeCO_{2}\right)/\left(1-FiO_{2}-FiCO_{2}\right)-FeO_{2}\right) \end{array}$$

(6) $$\begin{array}{l} VCO_{2}\;\mathrm{produced}=Qe^{*}\;\left(FeCO_{2}-FiC{O_{2}}^{*}\left(FeN_{2}/FiN_{2}\right)\right)\\ =Qe^{*}\;\left(FeCO_{2}-FiC{O_{2}}^{*}\left(1-FeO_{2}-FeCO_{2}\right)/\left(1-FiO_{2}-FiCO_{2}\right)\right) \end{array}$$

If all the measurement of gas volume fractions are perfect, then the only source of error in this method of calculating VO_2_ and VCO_2_ is the error in measuring the exhale volumetric flow. Note also that the Haldane transform can also be applied to reference the calculations to the inhale volumetric flow. The CORVET data processing, employs the Haldane transform to calculate VO_2_ and VCO_2_ with reference to both the inhale and exhale flows. Whether referenced to inhale or exhale, the number of errors exceeding the threshold of 10% is similar and significantly lower than for the direct method, implying that inconsistency in the flow measurements between the inhale and exhale limb is the dominate source of error in the direct method.

The Haldane method reduces the errors due to inconsistencies (i.e., bias) between inhale and exhale flows. There is a variation on the Haldane method described above that attempts to reduce the impact of gas sensor errors by employing the same physical mixing chamber and gas sensors to measure the gas concentrations successively in time on both the inhale and exhale limb. This approach assumes that over brief periods of time, on the order of a few minutes, the average inhale and exhale flows and breath profiles will be consistent and so a single mixing chamber can be used to measure the gas concentrations on the inhale limb for a period of time and then switched to measure the gas concentrations on the exhale limb with the sequential measurements used in Equations 5 and 6 in place of concurrent measurements made by two separate mixing chambers. The benefit of this approach is that any bias (offset terms) in the gas sensor will tend to be canceled during the subtraction of the flows from the inhale and exhale limbs. This method of calculating VO_2_ and VCO_2_ is termed the *Common Mode Haldane* and, as with the traditional Haldane, can be referenced to either the inhale or exhale limb.

## 4 Ventilator test bed and prototype validation

**Figure 8:**
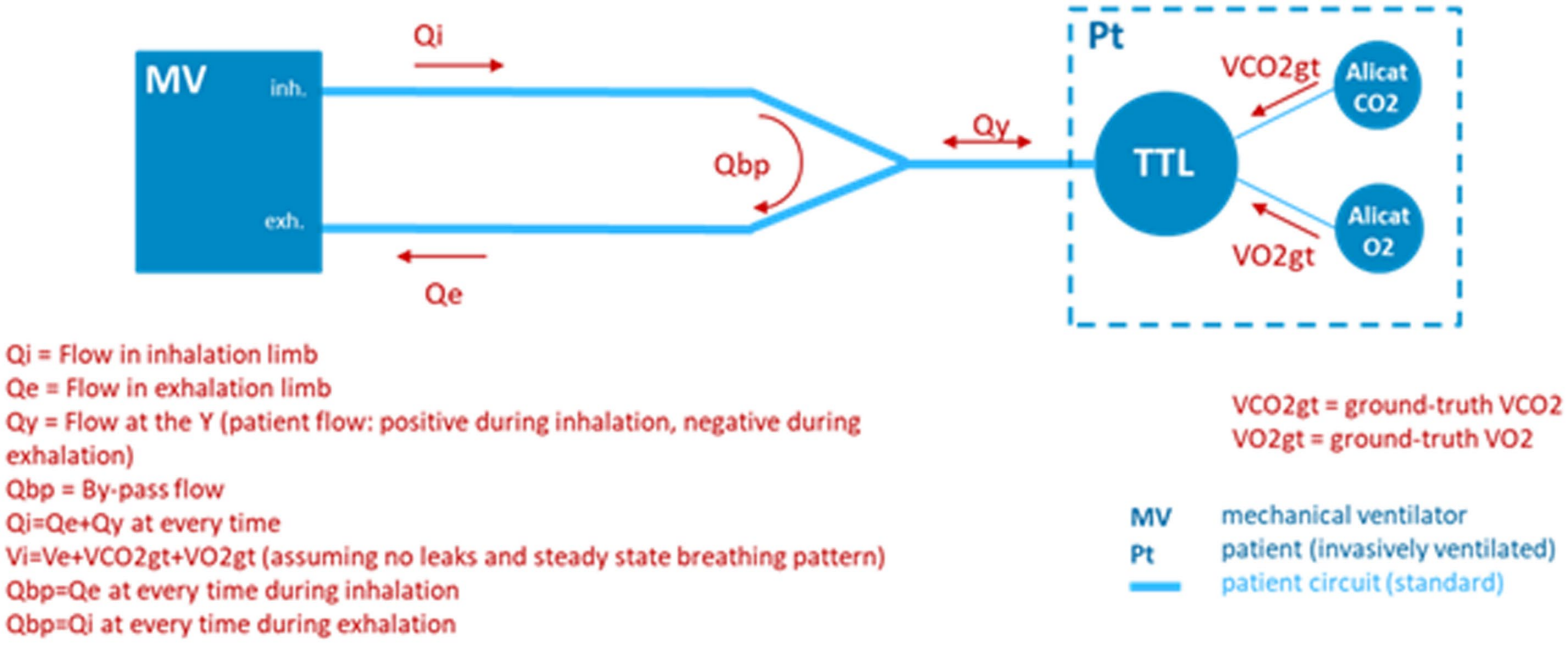
Test bed configuration employing a ventilator and test and training lung (TTL) to provide realistic breath profiles. In the absence of a precise Qi, ground truth differential volumetric flows are created by injecting known volume rates of O_2_ and CO_2_ into the TTL by means of mass flow controllers.

**Figure 9:**
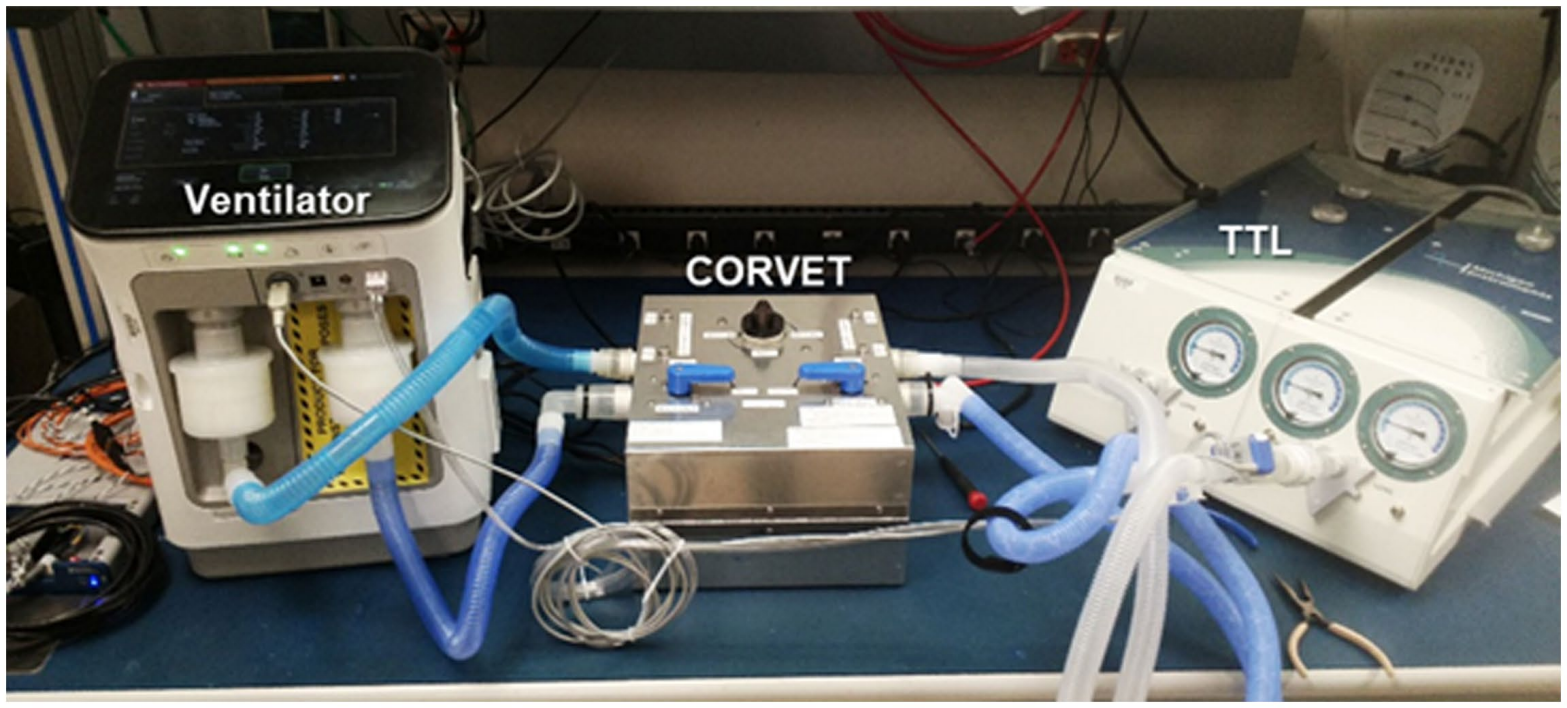
Ventilator, CORVET prototype, and test training lung comprising validation test bed.

In order to simulate the environment of a mechanically ventilated patient, but provide ground truth knowledge of exhale gas mixtures, a test bed was assembled incorporating a mechanical ventilator and a test training lung (TTL) (25), as indicated in the block diagram of Figure 8. A photo of the simulation test bed showing the ventilator, CORVET prototype and TTL connections is shown in Figure 9.

It was recognized that to validate performance of the CORVET in the simulation test bed, one cannot assume the ventilator flow settings and O_2_ fractional volumes are accurate, hence the ventilator settings cannot be used as a ground truth reference against which to compare the VO_2_ measurements of the CORVET prototype. In order to provide a non-zero, adjustable, measurable, and reliable ground truth VO_2_ reference, controlled injection of known amounts of CO_2_ and O_2_ into the TTL were made to simulate various respiratory exchange ratios, albeit with an increase, rather than depletion, of the exhaled VO_2_ by the simulated patient.

Increasing the VO_2_ in the exhale is counter to actual physiology but provides a reliable, measurable, ground truth reference that is not impacted by dilution or variation in ventilator flow from prescribed settings. However, injection of O_2_ into the TTL, rather than the depletion that would normally occur with a human subject, can lead to unrealistically small differences in the consumed O_2_, creating divide by zero singularities in some of the test cases. These singularities impose an unrealistic gas measurement accuracy that would not be present in the target application, since human metabolism consumes oxygen rather than generating it. In particular, while the injection of CO_2_ in the test lung effectively lowers the O_2_ concentration (from the initial FiO_2_), the injection (rather than depletion) of O_2_ raises the O_2_ volume concentration (rather than further decreasing it). Depending upon the rate of O_2_ and CO_2_ injection, the FeO_2_ concentration might be raised back to a value very close to the original FiO_2,_ requiring extremely precise O_2_ gas concentration measurement to capture the very small differences between FiO_2_ and FeO_2_ with sufficient accuracy. For the test cases in which this singularity occurs, suppression of the CO_2_ injection is employed to enable reasonable differences between FiO_2_ and FeO_2_ in determining the achievable VO_2_ measurement accuracy.

In a clinical setting, oxygen consumption, in concert with CO_2_ production, leads to an increased gap between FiO_2_ and FeO_2_, resulting in a more favorable differential signal to noise measurement.

## 5 Test cases and data collection protocols

As shown in Table 2, the test cases include a range of ventilator and TTL settings intended to simulate typical clinical conditions as well as a few extreme corner cases. The range of ventilator settings include respiration rate (RR), breath profiles (BP), including effects such as positive end expiatory pressure (PEEP), tidal volumes and oxygen concentrations. The range of TTL settings include resistance, simulating the respiratory impedance, compliance, simulating the lung elasticity, and tidal volume, simulating the exhale lung volume. Another ventilator parameter is blower mode versus compressed gas (CG). Blower mode is invoked only when the patient must be moved, which involves temporarily disconnecting from the sources of compressed air and oxygen and briefly supporting ventilation by means of a blower mixing ambient air with an oxygen source. Since the blower mode is only employed for a short period of time, the testing focused primarily on compressed gas test cases.

A collection of 30 test cases were explored with tidal volumes of 300, 500, or 800 mL, PEEPs of 5, 10, or 15 mbar, and respiration rates of 10, 20, or 30 BPM. The most relevant metric for accuracy of the metabolic sensor is the measured VO_2_ and VCO_2_. A number of test cases were run more than once, in part to assess the impact of sensor drift over time, and also to assess the impact of gas sensor recalibration that occurred at the beginning of March. As described in Section 4, the injection of both CO_2_ and O_2_ into the TTL creates some unrealistically small differences in FiO_2_ and FeO_2_ for a few of the test cases, in particular those with FiO_2_ in the range of 40–60%. These singular test cases require measuring gas concentration differences between FiO_2_ and FeO_2_ of less than 1% and, since the paramagnetic O_2_ sensor precision is specified as ±0.2%, absolute concentration error, the result is an increase in the VO_2_ relative percent measurement error that would not occur in the case of physiologically realistic O_2_ consumption. For this reason, a 1,000 series of test cases was created in which the CO_2_ injection was suppressed and the O_2_ injection increased in order to achieve differences between FiO_2_ and FeO_2_ that are realistic, apart from the sign (FeO_2_ > FiO_2_). Only the nominal VO_2_ and VCO_2_ were changed relative to the baseline case, with all other parameters preserved for the 1,000 series. The last 3 digits of the 1,000 series test cases indicate the corresponding baseline test case.

Table 2 summarizes the ventilator and TTL injection settings for the baseline test cases and represents the 1,000 series cases with the addition of a column showing the modification in O_2_ injection relative to the associated baseline test case. The data collects conducted in February represent 19 unique ventilator test cases of interest, first explored with dry air to obtain a baseline performance data set. The 13 data collects in March repeated some of the dry air baseline cases to assess consistency and the impact of recalibration of the gas sensors as well as investigating the impact of humidity on performance.

**Table 2:** Summary of baseline test case ventilator settings and associated ground-truth (gt) injection rates of CO_2_ and O_2_.

Date of data collect	Test case #	Breath profile	TTL resistance	TTL compliance	Dry/Humid	CG/Blower	PEEP	Ground truth values							
								O_2_	Nominal VE				ALICAT		VCO_2_ = 0
								FiO_2_%	RR BPM	VT L	Peak flow LPM	RR×VT LPM	VO_2_gt ml/min	VCO_2_gt ml/min	VO_2_gt
															ml/min
2/3	209	Square	20	0.05	Dry	CG	5	60	30	0.3	73	9	400	400	1,000
2/3	207	Ramp	20	0.02	Dry	CG	5	21	10	**0.8**	**170**	8	300	300	400
2/3	13	Square	20	0.05	Dry	CG	10	40	20	0.5	87	10	400	400	700
2/3	9	Square	20	0.02	Dry	CG	5	30	20	0.5	72	10	300	300	450
2/1	5	Square	20	0.02	Dry	CG	10	21	20	0.5	146	10	300	300	400
2/3	7	Ramp	20	0.02	Dry	CG	10	30	20	0.5	134	10	300	300	450
2/3	11	Ramp	20	0.02	Dry	CG	5	30	20	0.3	65	6	300	300	450
2/3	15	Ramp	20	0.05	Dry	CG	10	40	20	0.5	73	10	400	400	700
2/17	1	Square	5	0.05	Dry	CG	5	21	20	0.5	49	10	250	300	350
2/17	3	Ramp	5	0.05	Dry	CG	5	30	20	0.5	71	10	250	300	400
2/17	204	Ramp	5	0.05	Dry	CG	5	60	10	**0.8**	**71**	8	250	300	750
2/17	206	Square	5	0.05	Dry	CG	5	50	30	0.3	33	9	250	300	600
2/17	17	Square	5	0.02	Dry	CG	5	40	20	0.5	119	10	430	300	700
2/17	19	Ramp	5	0.02	Dry	CG	5	60	20	0.5	112	10	430	300	1,000
2/17	201	Ramp	5	0.02	Dry	CG	15	60	20	**0.8**	**192**	16	430	300	1,000
2/22	2	Square	5	0.05	Dry	Blower	5	30	20	0.5	64	10	250	300	350
2/22	12	Ramp	20	0.02	Dry	Blower	5	21	20	0.3	74	6	300	300	450
2/22	16	Ramp	20	0.05	Dry	Blower	10	40	20	0.5	88	10	400	400	700
2/22	203	Square	5	0.02	Dry	Blower	5	60	30	0.3	85	9	430	300	600
3/1	2	Square	5	0.05	Dry	Blower	5	21	20	0.5	64	10	250	300	350
3/1	12	Ramp	20	0.02	Dry	Blower	5	30	20	0.3	74	6	300	300	450
3/8	209	Square	20	0.05	Humid	CG	5	60	30	0.3	73	9	400	400	1,000
3/8	207	Ramp	20	0.02	Humid	CG	5	21	10	**0.8**	**170**	8	300	300	400
3/8	17	Square	5	0.02	Humid	CG	5	40	20	0.5	119	10	430	300	700
3/8	1	Square	5	0.05	Humid	CG	5	21	20	0.5	49	10	250	300	350
3/29	1	Square	5	0.05	Dry	CG	5	21	20	0.5	49	10	250	300	
3/29	5	Square	20	0.02	Dry	CG	10	21	20	0.5	146	10	300	300	
3/29	11	Ramp	20	0.02	Dry	CG	5	30	20	0.3	65	6	300	300	
3/31	15	Ramp	20	0.05	Dry	CG	10	40	20	0.5	73	10	400	400	
3/31	17	Square	5	0.02	Dry	CG	5	40	20	0.5	119	10	430	300	
3/31	207	Ramp	20	0.02	Dry	CG	5	21	10	**0.8**	**170**	8	300	300	
3/31	209	Square	20	0.05	Dry	CG	5	60	30	0.3	73	9	400	400	

The right-most shaded column highlights the only difference between the baseline and 1,000 series test cases, in which CO_2_ injection is suppressed and O_2_ injection rate is modified as indicated. Note that the FiO_2_ for test case 2 was reduced from 30% in the February collection to 21% during the March collection. The exceptionally high tidal volume (0.8 L) for a ventilated subject is highlighted in blue font.

In addition to the range of ventilator and TTL settings represented by each test case, there is also the option to perform testing with the dry compressed gases, which eliminates the confound of humidity, or to include a humidifier on the input limb of the ventilator, which is necessary for actual ventilation of either animals or humans. For reasons of efficiency and reducing the number of variables affecting accuracy, initial testing was performed using dry gases. A subset of the test cases were then re-run employing a humidifier which heats the inspired gas to the range of 37–39C and saturates it with water vapor. The water vapor changes the overall volume fraction of the O_2_, N_2_, and CO_2_ gases on inhale but is measured and accounted for in converting the measured volume concentrations to STPD conditions. The major challenge associated with humidification is ensuring the saturated gas does not condense on the gas sensor optical surfaces in the mixing chambers, which would seriously degrade measurement accuracy as well as the lifetime of the sensors. This is accomplished using a Nafion^®^ dryer which equilibrates the ~1% side-stream sample of the humidified inhale or exhale air to nominal room temperature and humidity before it enters the mixing chamber. Temperature and humidity sensors in the mixing chamber measure the RH and T associated with the gas volume enabling conversion to standard temperature, pressure, and dry air (STPD) conditions.

For each test case, data is first collected in Configuration A, in which mixing chamber 1 is connected to the inhale limb, prior to the humidifier, and mixing chamber 2 is connected to the exhale limb as illustrated in Figure 6. Data is collected in this configuration for a sufficient period of time, nominally 150 breaths, to ensure evacuation of residual ambient air or gas mixtures from previous testing from the sample tubes and mixing chambers resulting in a steady state within each mixing chamber. A port swap is then executed, effectively reversing the roles of the two mixing chambers, placing mixing chamber 2 on the inhale limb and mixing chamber 1 on the exhale limb (Configuration B).

As described in section 3.2, a benefit of the port swapping is to investigate the potential for reducing or eliminating the impact of gas calibration drift, in particular an offset bias in the gas sensor measurements, by using the data collected by the same mixing chamber in Configuration A, with data collected at a later time in Configuration B. The time-delayed data collected with the same mixing chamber can then be used to compute the gas volume concentrations necessary to produce the VO_2_ and VCO_2_ needed to determine RER and energy expenditure. Provided the ventilator settings are not changed during the port swap, and the patient metabolism is not rapidly fluctuating, an offset or bias in either the O_2_ or CO_2_ sensor will degrade gas volume measurements on the inhale and exhale limbs in the same manner and hence, subtracting the average measurements made by the same mixing chamber at two different times corresponding to configuration A and configuration B should, in theory, cancel the bias term. This approach is termed the *common-mode rejection* scheme.

## 6 CORVET performance analysis and characterization

A summary and discussion of the CORVET prototype performance is presented in this section, predominantly for the dry, compressed gas test cases. A sampling of humidified and blower-only test cases is included as well.

### 6.1 Principle performance metrics

The critical performance metric is the accuracy of the measured VO_2_ and VCO_2_ since these quantities are the basis for determining both the RER and energy expenditure of the ventilated patient. The accuracy of the VO_2_ and VCO_2_ is directly impacted by the accuracy of the rate-proportional side-stream sampling, the gas concentration measurements, and the volume flow measurement.

### 6.2 CORVET acceptable error threshold

Given the prototype nature of the CORVET, incorporation of gas sensors with a precision of no better than 0.2% absolute, and a COTS flow sensor with errors as high as 25% at flow rates approaching 200LPM, we set an acceptable measurement error goal of 10% or less for the measurement of VO_2_ and VCO_2_ with the understanding that errors could be reduced in a design for manufacture system by incorporation a more accurate flow sensor. To understand how a 10% measurement error in volume flow impacts the key metabolic parameters of RER (fuel substrate) and energy expenditure, consider nominal volumetric flows and an assumed RQ of 1. Since many of the test cases involve VO_2_ and VCO_2_ flows of 300 mL/min, in the worst case, an under-estimate of O_2_ consumption by 10% and over-estimate of CO_2_ production by 10% would translate to an RER of 330/270 = 1.22 or 22%. This is not an insignificant error since RQ >1 implies *de novo* lipogenesis activity. However, if the errors were both in the same direction, there would be no error in the estimate of RER. In any case, a completely erroneous error in estimating RER (e.g., 1 versus 0.7), results in no more than a 7% error in the estimate of energy expenditure from the Weir equation. However, the error in measuring the volume flow rate of gas transfers directly to an error in energy estimation. In particular, a 10% error in estimating flow results in a 10% error in estimating VO2, which in turn results in a 10% error in estimating energy expenditure, assuming the gas concentration measurements are correct.

As further justification of a 10% or better accuracy goal, note also that over the years, a number of studies have been conducted comparing the agreement between various gold-standard metabolic carts (26–28) with observed differences typically in the 10% range and in some cases as high as 20%. The implication is that accuracy better than 10% is difficult to achieve even for high-end, gold-standard metabolic carts employing multi-liter mixing chambers and calibration occurring just prior to the time of the comparison.

### 6.3 CORVET measured performance across baseline test cases

With this background, Table 3 provides a summary of the performance of the CORVET prototype over the range of test cases. Errors in VO_2_ and VCO_2_ exceeding 10% are highlighted in red font. The VO_2_ and VCO_2_ measurements presented in Table 3 are obtained by employing a Haldane transform with flows referenced to the exhale limb. Port swapping was performed to enable calculation by means of common-mode processing and averaging the measured values from both Configuration A and Configuration B. After discussing the performance obtained using the single-sided Haldane transform, comparison with the other processing modes is presented.

**Table 3:** Summary of CORVET VO_2_ and VCO_2_ measurement accuracy for 19 baseline test cases, 7 repeats plus variations examining impact of blower, humidity, and no CO_2_ injection on performance.

Date of data collect	Dry/Humid	CG/Blower	Ground truth values								Measured with MITLL flow cal				
			O_2_	Nominal VE				ALICAT		VCO_2_ = 0	CORVET		% Error	CO_2_ ≠ 0	CO_2_ = 0
			FiO_2_	RR	VT	Peak	RR×VT	VO_2_gt	VCO_2_gt	VO_2_gt	VO_2_	VCO_2_	ΔVCO_2_	ΔVO_2_	ΔVO_2_
			%	BPM	L	LPM	LPM	ml/min	ml/min	ml/min	L/min	L/min	%	%	%
2/3	Dry	CG	60	30	0.3	73	9	400	400	1,000	0.484	0.399	−0.2	21.1	0.2
2/3	Dry	CG	21	10	**0.8**	**170**	8	300	300	400	0.375	0.385	28.3	25.0	17.9
2/3	Dry	CG	40	20	0.5	87	10	400	400	700	0.458	0.455	13.8	14.4	5.7
2/3	Dry	CG	30	20	0.5	72	10	300	300	450	0.302	0.303	1.0	0.5	−4.2
2/1	Dry	CG	21	20	0.5	146	10	300	300	400	0.290	0.278	−7.5	−3.4	−6.1
2/3	Dry	CG	30	20	0.5	134	10	300	300	450	0.282	0.277	−7.7	−6.0	−7.2
2/3	Dry	CG	30	20	0.3	65	6	300	300	450	0.302	0.307	2.5	0.8	−5.0
2/3	Dry	CG	40	20	0.5	73	10	400	400	700	0.464	0.456	14.0	16.1	7.2
2/17	Dry	CG	21	20	0.5	49	10	250	300	350	0.263	0.320	6.5	5.3	1.8
2/17	Dry	CG	30	20	0.5	71	10	250	300	400	0.264	0.318	5.9	5.5	2.1
2/17	Dry	CG	60	10	**0.8**	**170**	8	250	300	750	0.290	0.310	3.5	16.0	2.7
2/17	Dry	CG	50	30	0.3	33	9	250	300	600	0.266	0.300	−0.1	6.3	−2.8
2/17	Dry	CG	40	20	0.5	119	10	430	300	700	0.441	0.304	1.2	2.5	1.2
2/17	Dry	CG	60	20	0.5	112	10	430	300	1,000	0.441	0.290	−3.3	2.6	1.5
2/17	Dry	CG	60	20	**0.8**	**192**	16	430	300	1,000	0.568	0.354	18.0	32.2	28.3
2/22	Dry	Blower	30	20	0.5	64	10	250	300	350	0.249	0.313	4.2	−0.5	1.9
2/22	Dry	Blower	21	20	0.3	74	6	300	300	450	0.318	0.315	5.0	5.9	2.1
2/22	Dry	Blower	40	20	0.5	88	10	400	400	700	0.393	0.407	1.7	−1.9	−0.7
2/22	Dry	Blower	60	30	0.3	85	9	430	300	600	0.470	0.293	−2.2	9.3	12.4
3/1	Dry	Blower	21	20	0.5	64	10	250	300	350	0.263	0.314	4.5	5.0	1.9
3/1	Dry	Blower	30	20	0.3	74	6	300	300	450	0.301	0.311	3.5	0.4	2.1
3/8	Humid	CG	60	30	0.3	73	9	400	400	1,000	0.463	0.416	4.0	15.7	9.0
3/8	Humid	CG	21	10	**0.8**	**170**	8	300	300	400	0.362	0.382	27.3	20.6	17.0
3/8	Humid	CG	40	20	0.5	119	10	430	300	700	0.474	0.327	9.0	10.2	4.0
3/8	Humid	CG	21	20	0.5	49	10	250	300	350	0.265	0.322	7.4	6.1	0.3
3/29	Dry	CG	21	20	0.5	49	10	250	300		0.256	0.281	−6.3	2.3	
3/29	Dry	CG	21	20	0.5	146	10	300	300		0.262	0.230	−23.4	−12.8	
3/29	Dry	CG	30	20	0.3	65	6	300	300		0.298	0.279	−7.1	−0.5	
3/31	Dry	CG	40	20	0.5	73	10	400	400		0.409	0.385	−3.7	2.3	
3/31	Dry	CG	40	20	0.5	119	10	430	300		0.419	0.256	−14.8	−2.5	
3/31	Dry	CG	21	10	**0.8**	**170**	8	300	300		0.364	0.335	11.8	21.5	
3/31	Dry	CG	60	30	0.3	73	9	400	400		0.429	0.381	−4.7	7.3	

The blue columns are the baseline testing without suppression of CO_2_ and the green columns show th modified ml/min of O_2_ when CO_2_ is suppressed and the resulting improvement in error. Errors in VO_2_ and VCO_2_ exceeding 10% are highlighted in red font. The exceptionally high tidal volume (0.8 L) for a ventilated subject is highlighted in blue font.

#### 6.3.1 February baseline test cases

The first 19 rows of Table 3, encompassing all of the February tests, are dry baseline test cases that include 6 blower cases. The ventilator supplied O_2_ concentrations range from 21 to 60% and the tidal volumes range from 0.3 to 0.8 L, with 0.8 L an exceptionally high tidal volume for a ventilated subject and highlighted in Table 3 with blue font. Respiration rates varied from 10 breaths per minute to a high of 30 breaths per minute. The ground truth reference gas injections, denoted by column headings VO_2_gt and VCO_2_gt, ranged from a low of 250 mL/min to a high of 430 mL/min. The gas sensors were recalibrated between the February and March runs, so we first review the performance observed during the February collects.

The volume flows measured by the CORVET are shown in the blue shaded column. These flows were obtained by applying a Lincoln Laboratory derived flow calibration to the COTS flow sensor. For the first 19 baseline test case entries in Table 3, four of the VCO_2_ measurements, and six of the VO_2_ measurements exceeded the 10% error performance threshold for a total of 10 exceedances out of 38 measurements implying 26% of the measured flows were unacceptable.

However, four of the error exceedances correspond to test cases with a tidal volume of 0.8 L and a respiration rate, PEEP and lung compliance that result in peak flow rates of 170LPM or more. The specifications for the COTS flow sensor employed in the CORVET prototype indicate that at flow rates of 170LPM or more flow errors might be 25% or higher. This suggests that the volume flow errors associated with the 0.8 L high peak flow cases are likely attributable to inconsistency in the COTS flow sensor at high flow rates. Procurement or development of a higher dynamic range flow sensor should be considered in any future testing or design for manufacture.

As further evidence for the assertion that high peak flow leads to larger flow sensor errors, note that test cases 3, 7 and 9 have the same FiO_2_, RR, and VT but the peak flow rates are significantly higher for TC7 due primarily to a lower lung compliance setting and, to a lesser degree, a different waveform profile (square vs. ramp). As indicated in Table 3, test cases 3 and 9 produce smaller errors than TC7.

In similar fashion, note that test cases 1 and 5 have the same FiO_2_, RR, VT and breath profile but differ in compliance and resistance resulting in a peak flow difference of nearly 3X with a peak flow of 146LPM for TC1 and a larger magnitude error in VCO_2_. Taking into account, along with the sign change in the error between TC1 and TC5, there is more than a 10% difference between TC1 and TC5, reinforcing the claim that variability of the COTS flow sensors at high peak flows is a significant source of error. The COTS flow sensor employs a grid structure to enforce laminar flow and assumes laminar flow in the flow rate computation. We conjecture that turbulent flow induced by the high peak flows is likely responsible for the inaccuracy of the reported flow. Additional testing to further validate our conjectures can be beneficial if the device is intended to be used at high tidal volume and high exhalation peak flow. If we attribute the errors in the 0.8 L tidal volume cases to the variance in the COTS sensor at high peak flows, for the remaining 4 VO_2_ baseline test case exhibiting measurement errors that exceed 10% (test cases 13, 15, 204, 209), all of them correspond to cases in which the fraction of inspired oxygen, FiO_2_, is either 40 or 60% and the volume rates of the O_2_ and CO_2_ injected into the lung are the same or nearly the same. As previously noted, artificially injecting O_2_ into the lung, rather than depleting it, tends to offset the reduction in O_2_ volume fraction resulting from exhaled CO_2_, bringing the exhaled O_2_ concentration, FeO_2_ closer to the inspired value. As a result, unrealistically high precision in the gas sensor is required to measure the very small differences between the inspired and expired O_2_ concentrations with less than a 10% error. In particular, for identical injected O_2_ and CO_2_ volume rates, the 0.2% precision of the oxygen sensor is insufficient to produce accurate VO_2_ when the FiO_2_ is in the range of 40–60%. The same test cases 13, 15, 204, 209 run with higher VO_2_gt and zero VCO_2_gt to increase the difference between FiO_2_ and FeO_2_ to realistic values (except for the sign), yield lower errors, all within the desired 10% limit. Discounting these cases along with the 0.8 L tidal volume cases, there are no other VO_2_ errors exceeding 10% and only two VCO_2_ errors, test case 13 with a VCO_2_ error of 13.8% and test case 15 with a VCO_2_ error of 14.0%, corresponding to just 5.2% of the total set of 38 baseline measurements.

#### 6.3.2 No CO_2_ injection February baseline test cases

In order to quantify the impact of the artificial injection, rather than depletion, of O_2_ in the baseline test cases, a 1,000 series of test cases was created in which CO_2_ injection was suppressed and O_2_ injection increased with no change to any other test case parameters in order to assess the impact of more realistic O_2_ concentration gradients (absolute difference between FiO_2_ and FeO_2_). With reference to Table 3, the two green columns correspond to 1,000 series test cases in which no CO_2_ was injected, and the O_2_ ground truth volume flows were modified as shown. The last column in Table 3 shows the VO_2_ error for the corresponding baseline test case when CO_2_ injection is suppressed,

With reference to Table 3, in the first 19 rows, corresponding to the baseline test cases, when CO_2_ injection was suppressed, only three VO_2_ errors exceed 10% and two of the three exceedances occur for the 0.8 L tidal volumes with high peak flows. Discounting as before, the 0.8 L cases, test case 1,203 (blower), with an error of 12.4%, is the only case in which the VO_2_ error exceeded the 10% error threshold corresponding to an error exceedance rate of 1 in 17 or 5.9% of the cases.

#### 6.3.3 Repeat of the baseline test cases in March

Referring again to Table 3, the data collections in March are effectively repeats of the earlier baseline test cases after adding Nafion^®^ dryers to enable testing with humidified air. Test cases 1, 17, 207 and 209 were repeated with heated and saturated air and a second round of testing was completed with dry air for comparison to the February test data. In addition to the inclusion of the Nafion^®^ dryers, the gas sensors were also recalibrated and the flow dividers were re-oriented to prevent any condensation from entering the sample lines. As indicated by the data in Table 3, heating and humidifying the inspired gas did not seem to increase the error rate, and the trends in the March data were similar to those observed in the February data. Discounting the 0.8 L high peak flow cases, there were two VCO_2_ measurements that exhibited more than a 10% error and three VO_2_ measurements exhibiting larger than a 10% error, two of which were eliminated by suppressing the CO_2_ injection for reasons previously described. Consequently, the total number of otherwise unexplained exceedances was 3 out of 22 or 13.6% of the volume flow measurements. Two of these exceedances were for test case 5 with a relatively high peak flow rate of 146 LPM. Discounting the 0.8 L cases, the average VO2 error for combined series 1,000 cases in February and March was 1.3%, with a standard deviation of 4.8% and the average VCO_2_ error was 3.0% with a standard deviation of 5.7%.

#### 6.3.4 Alternative data processing algorithms

The data in Table 3 was obtained by employing the average of single Haldane transforms referenced to the exhale limb, along with an MIT Lincoln Laboratory custom flow calibration. In Table 4, a comparison of the three processing algorithms described in section 3.3 is presented. Recall the three algorithms are the Direct method, the Haldane referenced to exhale, and the Common Mode Haldane referenced to exhale. To further reduce the dimensionality of the data, the entries in Table 4 represent the average of the measured values in the initial mixing chamber locations (configuration A) versus the swapped locations (configuration B).

**Table 4:** Comparison of VO_2_ and VCO_2_ measurement accuracy for Direct, Haldane, and Common Mode algorithms with MITLL custom flow calibration applied.

Test case	Measured		Ground truth		Direct			Common mode exhale					Single exhale Haldane			
	O_2_	Peak	ALICAT		CORVET		% Error CO_2_ ≠ 0	CORVET			% Error CO2 ≠ 0		CORVET	% Error CO_2_ ≠ 0		
	FiO_2_	Flow	VO_2_	VCO_2_	VO_2_	VCO_2_	ΔVCO_2_	ΔVO_2_	VO_2_	VCO_2_	ΔVCO_2_	ΔVO_2_	VO_2_	VCO_2_	ΔVCO_2_	ΔVO_2_
#	%	LPM	ml/min	ml/min	L/min	L/min	%	%	L/min	L/min	%	%	L/min	L/min	%	%
209	60	73	400	400	0.777	0.399	−0.4	94.2	0.484	0.399	−0.2	21.1	0.484	0.399	−0.2	21.0
207	21	**170**	300	300	0.359	0.373	24.2	19.7	0.375	0.385	28.3	25.0	0.375	0.385	28.3	25.0
13	40	87	400	400	0.553	0.453	13.1	38.3	0.458	0.455	13.8	14.4	0.458	0.455	13.8	14.5
9	30	72	300	300	0.275	0.301	0.2	−8.2	0.302	0.303	1.0	0.5	0.302	0.303	1.0	0.5
5	21	146	300	300	0.398	0.276	−8.2	32.7	0.290	0.278	−7.5	−3.4	0.290	0.278	−7.5	−3.4
7	30	134	300	300	0.415	0.274	−8.6	38.5	0.282	0.277	−7.7	−6.0	0.282	0.277	−7.7	−6.0
11	30	65	300	300	0.270	0.304	1.3	−10.0	0.302	0.307	2.5	0.8	0.303	0.307	2.5	0.8
15	40	73	400	400	0.585	0.453	13.3	46.3	0.464	0.456	14.0	16.1	0.465	0.456	14.0	16.2
1	21	49	250	300	0.299	0.319	6.3	19.8	0.263	0.320	6.5	5.3	0.263	0.320	6.5	5.3
3	30	71	250	300	0.330	0.318	5.9	32.1	0.264	0.318	5.9	5.5	0.264	0.318	5.9	5.5
204	60	71	250	300	0.203	0.309	2.9	−18.6	0.290	0.310	3.5	16.0	0.290	0.310	3.5	16.0
206	50	33	250	300	0.318	0.299	−0.2	27.3	0.266	0.300	−0.1	6.3	0.266	0.300	−0.1	6.3
17	40	119	430	300	0.474	0.305	1.5	10.2	0.441	0.304	1.2	2.5	0.441	0.304	1.2	2.5
19	60	112	430	300	0.407	0.132	−56.1	−5.3	0.441	0.290	−3.3	2.6	0.440	0.290	−3.3	2.4
201	60	**192**	430	300	1.123	0.357	18.8	161.	0.568	0.354	18.0	32.2	0.569	0.354	18.0	32.3
2	30	64	250	300	0.137	0.314	4.6	−45.4	0.249	0.313	4.2	−0.5	0.249	0.313	4.2	−0.5
12	21	74	300	300	0.256	0.315	4.9	−14.6	0.318	0.315	5.0	5.9	0.318	0.315	5.0	5.9
16	40	88	400	400	0.261	0.403	0.7	−34.8	0.393	0.407	1.7	−1.9	0.392	0.407	1.7	−1.9
203	60	85	430	300	0.090	0.294	−1.9	−79.1	0.470	0.293	−2.2	9.3	0.470	0.293	−2.2	9.3
2	21	64	250	300	0.192	0.315	5.0	−23.2	0.263	0.314	4.5	5.0	0.263	0.314	4.5	5.1
12	30	74	300	300	0.209	0.310	3.3	−30.2	0.301	0.311	3.5	0.4	0.301	0.311	3.5	0.5
209	60	73	400	400	1.261	0.420	4.9	215.	0.463	0.416	4.0	15.7	0.462	0.416	4.0	15.5
207	21	**170**	300	300	0.513	0.379	26.4	71.1	0.362	0.382	27.3	20.6	0.362	0.382	27.3	20.6
17	40	119	430	300	0.763	0.328	9.2	77.4	0.474	0.327	9.0	10.2	0.474	0.327	9.0	10.2
1	21	49	250	300	0.562	0.174	−41.9	125.	0.265	0.322	7.4	6.1	0.265	0.322	7.4	6.1
1	21	49	250	300	0.318	0.279	−6.9	27.3	0.256	0.281	−6.3	2.3	0.256	0.281	−6.3	2.3
5	21	146	300	300	0.395	0.230	−23.3	31.8	0.262	0.230	−23.4	−12.8	0.262	0.230	−23.4	−12.8
11	30	65	300	300	0.315	0.277	−7.5	4.9	0.298	0.279	−7.1	−0.5	0.298	0.279	−7.1	−0.5
15	40	73	400	400	0.617	0.384	−4.1	54.3	0.409	0.385	−3.7	2.3	0.409	0.385	−3.7	2.3
17	40	119	430	300	0.492	0.256	−14.7	14.4	0.419	0.256	−14.8	−2.5	0.419	0.256	−14.7	−2.5
207	21	**170**	300	300	0.370	0.331	10.2	23.2	0.364	0.335	11.8	21.5	0.364	0.335	11.8	21.5
209	60	73	400	400	0.826	0.382	−4.5	106.	0.429	0.381	−4.7	7.3	0.429	0.381	−4.7	7.3

The three colors correspond to the test results for the three algorithm variations, Direct, Common Mode, and Single Haldane. Errors in VO_2_ and VCO_2_ exceeding 10% are highlighted in red font.

Several inferences can be gleaned from the percent errors reported in Table 4. Examining the performance of the Direct method, the VO_2_ errors are consistently worse than the VCO_2_ errors. Since the inhale CO_2_ concentration is nominally zero, the VCO_2_ error in the Direct method reflects the product of the exhale flow error and the exhale gas concentration error. However, since O_2_ concentration on inhale is large, the VO_2_ error reflects the errors and inconsistencies in the gas and flow measurements on both inhale and exhale. As noted in section 5, for FiO_2_ in the range of 40–60%, the injection of both O_2_ and CO_2_ requires very precise gas measurement to achieve less than 10% error in VO_2_ and that fact, along with the flow errors at high peak flow, result in a much higher number of VO_2_ errors above 10% than VCO_2_ errors.

Turning to the single and common-mode Haldane, Table 4 reveals there is very little difference in performance of the two algorithms across the test cases. The common mode Haldane, with sequential sampling in time of both inhale and exhale limbs by a single mixing chamber, is designed to reduce the impact of bias errors in the gas sensors. The fact that the single mode Haldane error performance is very similar to the common mode Haldane suggests that the gas sensors were well calibrated and any bias due to drift over the course of the experiments was insignificant.

## 7 Summary and conclusion

A ventilator and test training lung (TTL) were used to create a test bed capable of simulating a ventilated patient with variable tidal volume, respiration rate, impedance and compliance. Nineteen baseline test cases were defined to explore performance of the CORVET prototype over a range of O_2_ concentrations, respiration rates, ventilator waveforms and tidal volumes. In the course of testing, it was discovered that injection of both CO_2_ and O_2_ into the test lung to provide ground-truth simulated exhale gas mixtures created unrealistically small changes in the fractional volume of FeO_2_ when the input FiO_2_ was in the range of ~40–60%. The test cases were repeated with no CO_2_ injection and modified rates of O_2_ injection to obtain a more representative indication of the achievable VO_2_ measurement accuracy over the range of expected physiological O_2_ consumption. Seven of the 19 baseline test cases were repeated, some with heated and saturated input and others serving as a consistency check. In all, data was collected for 32 test cases plus an additional 25 test cases in which CO_2_ injection was suppressed and O_2_ injection increased, resulting in a total of 57 test cases analyzed.

In analyzing CORVET performance across the 57 test cases, it was observed that certain combinations of tidal volume, respiration rate and lung compliance resulted in peak flows of 170LPM or more and the test cases corresponding to these high peak flows generally resulted in errors larger than the desired 10% maximum. Since the large error was encountered on all of the 170LPM test cases, regardless of the simulated gas mixtures, the error is apparently due to the lack of accuracy in the commercial flow sensor employed in the prototype at flow rates above 100 LPM. Discounting these high peak flow cases, in the 19 baseline test cases there were 2 VCO_2_ errors and one VO_2_ error larger than 10% resulting in a success rate of 35/38 = 92%. This success rate was sufficiently high to warrant advancing to the next step in validation, animal testing.

While the Common Mode Haldane did not seem to offer substantial improvement over the Single Haldane, in a clinical environment, with days or weeks of continuous ventilation, drift may occur and port swapping provides an important mechanism to both confirm gas sensor calibration and enable cancelation of bias terms by rapid port swapping to obtain inhale and exhale data with the same mixing chamber.

### 7.1 Design-for-manufacture considerations

In a DFM implementation, a single mixing chamber located next to the ventilator on the exhale limb, along with a method for measuring FiO_2_ on the inhale limb prior to humidification, is capable of providing VO_2_ and VCO_2_ measurement with errors less than 10% with two provisions:

1. The flow sensor must be capable of providing accurate measurements at peak flows of 170LPM or more.
2. The gas sensor measurements must be good to 0.2% absolute or better and stable over the duration of the ventilation period.

The flow sensor requirement can be met either by procuring a better flow sensor than the COTS sensor used in the prototype, or by designing a better flow sensor and calibration process. Additional testing is needed to discover whether or not the inaccuracy of the flow sensor at high flows is due to failure of the design to ensure laminar flow at high peak rates. Since the algorithm for converting differential pressure to flow assumes a linear flow regime, it is possible that the transition to turbulent flow at high peak rates is the primary cause of the loss of accuracy at high peak flows.

The gas sensor calibration requirement is challenging but can be achieved with sensors costing a few hundred dollars in unit quantities. Since the O_2_ sensor is the more expensive of the two gas sensors, employing the same mixing chamber design, including the CO_2_ sensor, may be the most cost-effective way of measuring FiO_2_ on the inhale limb since only one mixing chamber design and calibration process is required for either the inhale limb or exhale limb sensors. Alternatively, active sampling or a similar concept could also be employed to measure O_2_ concentration on the inhale limb. The 2 months of data collection with the test bed did not reveal substantial problems with drift, but more extensive testing with animals and humans is needed to ensure calibration stability over longer periods of use in high humidity.

Full port swapping with computer control of the three and four-way valves will likely require a custom ball valve designed for the ventilator application. Port swapping provides a mechanism for detecting gas calibration drift with machine algorithms and without disrupting patient ventilation. Port swapping combined with common-mode processing also provides the capability to cancel bias in the gas sensors.

## Data availability statement

The original contributions presented in the study are included in the article/supplementary material, further inquiries can be directed to the corresponding author.

## Author contributions

GS: Conceptualization, Data curation, Formal analysis, Methodology, Project administration, Supervision, Validation, Visualization, Writing – original draft. FV: Conceptualization, Methodology, Resources, Supervision, Writing – review & editing. RB: Methodology, Validation, Visualization, Writing – review & editing.
